# Activation of a Raw Clay by Mechanochemical Process—Effects of Various Parameters on the Process Efficiency and Cementitious Properties

**DOI:** 10.3390/ma11101860

**Published:** 2018-09-29

**Authors:** Ilda Tole, Karin Habermehl-Cwirzen, Magdalena Rajczakowska, Andrzej Cwirzen

**Affiliations:** Department of Civil, Environmental and Natural Resources Engineering, Division of Structural and Fire Engineering, Luleå Tekniska Universitet, 97187 Luleå, Sweden; magdalena.rajczakowska@ltu.se

**Keywords:** clay minerals, dry grinding, fine grinding, mechanochemical activation, mechanochemistry, wet grinding

## Abstract

The efficiency of the mechanochemical activation (MCA) is influenced by various process parameters as well as by the properties of the treated material. The main objective of this research was to optimize the MCA process, gaining enhancement of the chemical reactivity of a Swedish raw clay, which is going to be used as an alkali-activated cementitious binder. The effects of the amount of water, the filling ratio, the rotation speed, and the grinding duration on the amorphization degree were evaluated by X-Ray Diffraction (XRD) and Scanning Electron Microscopy (SEM). Generally, wet and dry processes showed an extensive amorphization of both kaolinite and muscovite minerals present in the studied clay. On the contrary, quartz was amorphized mainly by the wet grinding process. The efficiency of both dry and wet grinding processes was enhanced by the increased number of grinding media versus the amount of the activated material. However, longer processing times caused significant agglomeration while a higher rotational speed enhanced the amorphization. Preliminary tests have shown that alkali activation of the processed clays produced hardened samples. Furthermore, the increased amorphization corresponded to the increased compressive strength values.

## 1. Introduction

The grindability of any material can be briefly described as an increase of the specific surface area per unit of the applied grinding energy [1]. While the grinding process can be divided, depending on the target particle size distribution, into coarse, intermediate, and fine grinding [2]. Mechanical properties of the processed material, including hardness, toughness, elasticity, and plasticity, but also the process regime, interaction phenomena, and environmental conditions, all strongly affect the grinding process [1,3,4]. The mechanochemical activation (MCA) is a special type of grinding process aiming not only to decrease the particle size distribution but also to induce structural changes, amorphization, and thus to increase the chemical reactivity of the processed material. The MCA process is commonly used in the mining industry, food processing, or pharmaceutics and chemicals production [5]. It is considered an environmentally friendly method because it avoids use of chemicals and high temperatures [2,6,7,8].

Fine grinding can be considered as a transitional state between a simple particle size reduction and the mechanochemical activation [4,7]. The MCA reactions occur due to lattice deformations, plastic deformations as well as due to a locally increased temperature caused by fracture and friction forces. A linear correlation between the specific surface area and the grinding duration was observed mainly in the initial stage of the process and was defined as the so-called Rittinger´s zone [4]. In the next stage, the produced effects include also enhancement of the chemical reactivity of the product. The early age stage of grinding provided the best conditions to optimize the onset time for effective mechanochemical reactions [4,9,10].

Various types of mills were used for the MCA, however, the application of a planetary ball mill proved to ensure sufficiently short processing times, safe handling and good reproducibility [1,8]. Planetary ball mills produced high acceleration forces, acting on the processed material through a combination of a rotational motion around the jar axis and a planetary motion around the main axis of the mill [11]. Several undesirable effects can occur during the MCA process, including for example agglomeration, caking effect or rolling movement of the balls [10,12,13]. The kinematic energy of the entire system can increase with a high ball to powder (B/P) ratio and with the increased jar rotation speed. An increased kinematic energy can lead to a more efficient process but at the same time, it can also result in caking, abrasion or contamination. Increased impact forces tend to produce finer powders but also increase the temperature of the processed material, which causes adhesion of the material to the inner surface of the jar. With the ongoing grinding process, the subsequent layers will start to build up and promote the caking effect, which eventually can decrease the efficiency of the entire process [14]. Using water or alcohol as a grinding medium can reduce the agglomeration and increase the efficiency of the process. At the same time, wet grinding can intensify shrinkage, cracking and deformations of the material exposed to the subsequent drying [4,10]. The dry grinding appeared to be more effective in imposing significant structural changes, even despite problems related to high temperature or agglomeration [4,15].

One example of a potentially wide application of the MCA process is the activation of clay minerals. The clay minerals are sustainable, commonly occurring and widely used in various applications including for example the construction sector. The calcinated kaolin, known as metakaolin, is used as a supplementary cementitious material (SCM) in the production of concrete or as a precursor for geopolymers [16,17,18]. High costs of the final product and requirements of high processing temperatures during the calcination have brought the attention to alternative processes. The MCA appeared as an environmentally friendly alternative excluding the need of high temperature, or chemical additives [7,8,19,20]. Earlier studies confirmed that the MCA is able to affect the structural order and the reactivity of such raw clay minerals as kaolinite, montmorillonite, illite and pyrophyllite [21,22,23]. Interestingly, the calcination process does not sufficiently affect the structural order of illite and montmorillonite but the MCA does [24].

The present study focused on determination of effects of various MCA process parameters on amorphization degree and chemical activation of a Swedish raw clay. A special emphasis was put on shortening of the processing time, minimization of contamination due to wearing of the grinding equipment and limiting agglomeration of the processed material. Additionally, preliminary tests were performed to verify the applicability of the processed clay as cementitious binders for geopolymer concrete.

## 2. Materials and Methods

**Figure 1:**
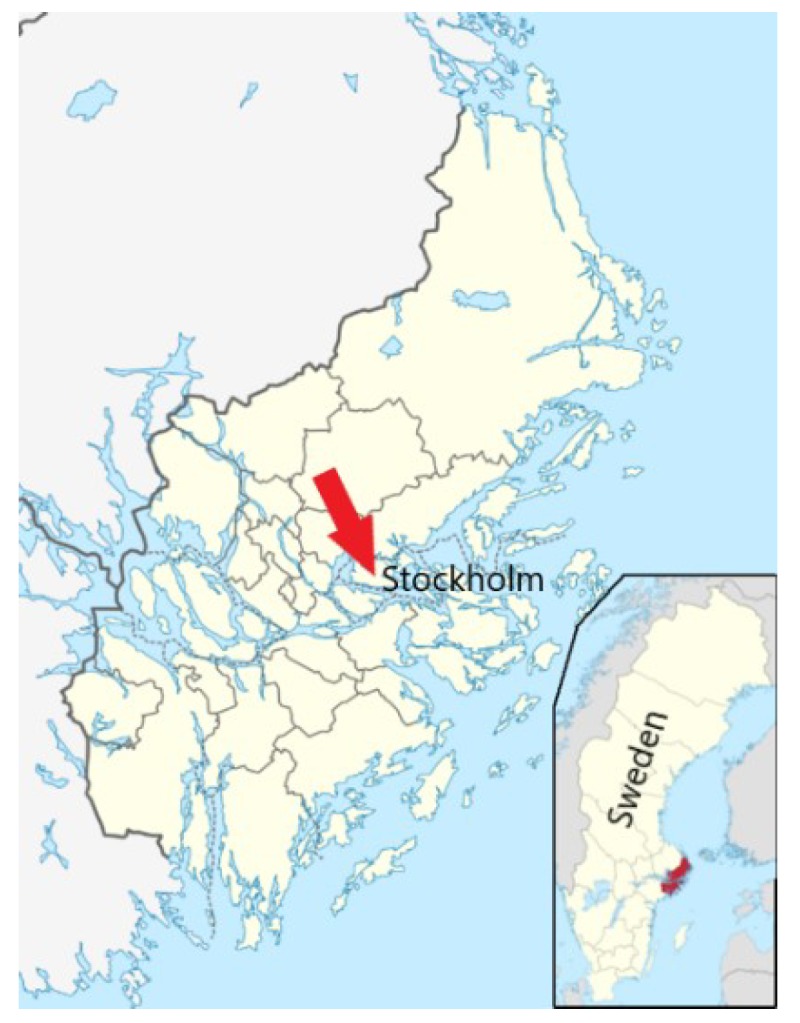
Sketch map of the region where the raw material was collected.

The raw material used in this study was collected near Stockholm in Sweden, Figure 1.

Geotechnical properties of the collected material were determined using the Atterberg limits. The obtained results showed values of 57% for the liquid limit, 23% for the plastic limit and the plasticity index of 34%. Sieve analysis on the raw material has indicated 51 % of clay, 31% of silt and 18% of sand, thus classifying the materials as clay with the standard soil texture triangle. The chemical composition of the used clay is shown in Table 1.

Clay samples were dried at 105 °C for 24 h, followed by a manual grinding. The unprocessed reference sample (S0) was also analyzed. Dry and wet grinding was done using a planetary ball mill, type Retsch PM 100 (Retsch GmbH, Haan, Germany), having a stainless steel jar with a volume of 500 mL. The used milling stainless steel balls had a diameter of 3 mm. Ball to powder mass ratio (B/P), water to powder mass ratio (W/P), the rotation speed and the duration of the grinding process were varied. The used process parameters are summarized in Table 2.

Changes of the crystallinity due to the MCA process were determined by the X-ray diffractometer type Empyrean from PANalytical with PIXcel 3D detector (Malvern Panalytical Ltd., Royston, UK), using Cu-Kα radiation with a wavelength of 1.54060 Å, generated at 45 kV and 40 mA. The step size was 0.0260 [°2θ] and the scan step size 87.4650 s. The temperature during the measurement was kept at 25 °C. The XRD samples were not pre-treated. Back-loading sample holders was used to avoid preferred orientation of the particles. Panalytical´s Highscore Plus equipped with a COD database determined the phase composition. The main identified phases include kaolinite (Kln), muscovite (Ms), illite (Ilt), montmorillonite (Mnt), quartz (Qz), and calcite (Cal). Origin 2018 software (OriginLab Corporation, Northampton, MA, USA) is used for calculation of the full width at half maximum (FWHM) of the kaolinite peak [001]. Lorentz fitting is used to assess the FWHM (001) indexes [25,26].

The particle surface morphology of raw and processed clays was observed by a Scanning Electron microscopy (SEM), type Jeol, JSM-IT100 (JEOL Nordic AB, Sollentuna, Sweden). High vacuum mode and a secondary electron detector (SED) were used. Images were taken from at least three. No conductive coating was applied. The particle size distribution and agglomeration were determined using a low vacuum mode and the backscattered electron detector (BEC). Dried clay samples were embedded into the resin under vacuum conditions and polished by using diamonds sprays with particle diameters of 9 μm, 3 μm and 1 μm. Nine images per sample were obtained at a magnification of 200 times. Subsequently, images were combined together with the use of the ImageJ software [27]. The analyzed surface was 1300 μm × 1300 μm. The spatial resolution of the images was 8 pixels per 1 μm allowing to observe a wide range of grain sizes as well as the agglomerated particles. The images were preprocessed with median filter with 2 pixel kernel to remove the noise. Afterwards, the threshold was adjusted manually to binarise the images, i.e., to segment the powder particles from the resin in the image. The analysis of the binarised images included determination of the basic morphological parameters, i.e., Feret diameter, perimeter, surface area, circularity index and equivalent circle diameter. Both image processing and calculation were performed with the use of ImageJ software [27,28,29,30]. The results were compared by fitting the Gaussian probability distributions to the histograms of selected morphological parameters.

Mortar mixes were used to determine the mechanical properties of alkali-activated processed clay samples. The sand to clay ratio was 1:1 in all tested mixes. The maximum particles size of the used sand was 0.15 mm. Sodium silicate provided by PQ Corporation (PQ Corporation, Valley Forge, PA, USA) was used as alkali activator (SS). The sodium silicate had the alkali modulus (Ms = SiO_2_/Na_2_O) by mass of 1, with 34.37 wt.% of SiO_2_ and Na_2_O, and solids content of 55.11 wt.%. The modulus of the sodium silicate solution was adjusted by the addition of sodium hydroxide pellets. The used amount of alkali activator was 10 wt.% by the mass of the processed clay in all mixes. Mix composition of the alkali-activated raw clay is shown in Table 3. 

All mixes were produced using a vacuum mixer, type Ecovac Bredent (Bredent GmbH & Co.KG, Senden, Gremany), with 290 rotations per minute. The mixing procedure included 1 min of mixing of all dry components followed by 2 min of wet mixing. Specimens had dimensions of 12 × 12 × 60 mm^3^ and were cast into molds made of Teflon, which excluded the usage of a demolding oil. Samples were removed from molds after 24 hours and were kept in sealed conditions until the compressive strength tests, which were done after 7-days. The compressive strength values were determined in a mechanical testing machine, type Instron model 1342. The tests were done on samples made using three types of clay; the untreated clay, (S0), the dry ground clay sample (DG-3R), and the most amorphized sample (DG-25R).

## 3. Results and Discussions

### 3.1. Grinding Media

**Figure 2:**
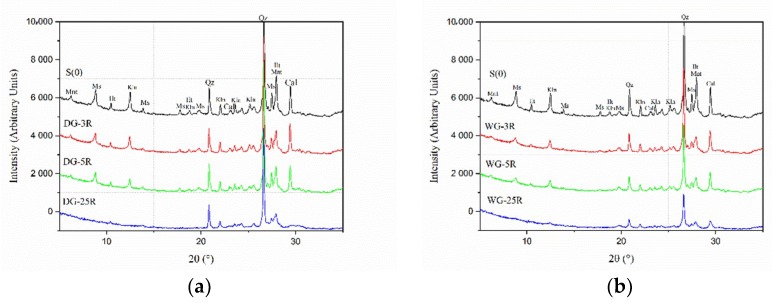
XRD diffraction patterns for different B/P ratio during grinding of a Swedish raw clay: (**a**) dry grinding process for samples S0, DG-3R, DG-5R and DG-25R; (**b**) wet grinding process for sample S0, WG-3R, WG-5R and WG-25R. (Kln-kaolinite, Mnt-Montmorillonite, Ms-muscovite, Ilt-illite, Qz-quartz, Cal-calcite).

**Figure 3:**
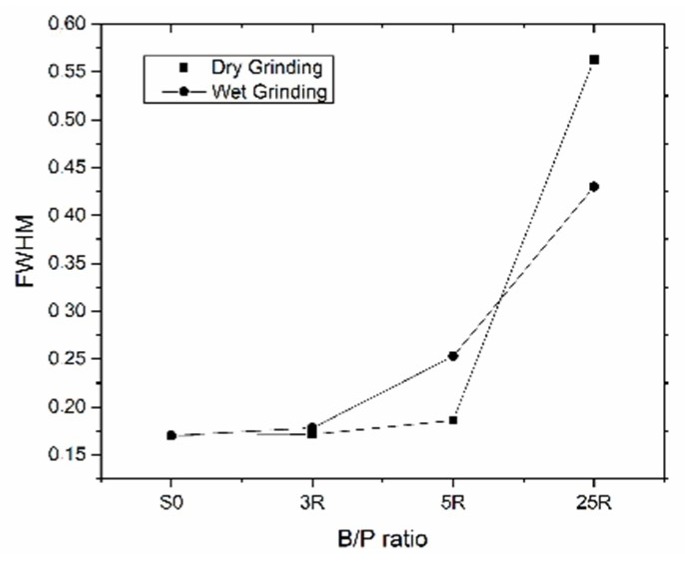
FWHM for kaolinite peaks [001] during dry and wet grinding with different B/P ratio.

In the first set of experiments, effects of the B/P ratio on the efficiency of the dry grinding process were studied. The following B/P ratios were used: 3:1 (DG-3R), 5:1 (DG-5R) and 25:1 (DG-25R), with a grinding time of 20 min. The XRD analysis were done for dry and wet ground materials, (Figure 2). The dry grinding process indicated a decrease in the crystallinity of the processed samples, (Figure 2a). The most extensive changes were observed for sample DG-25R, which had the B/P ratio of 25. Intensities of the peaks related to kaolinite (Kln), muscovite (Ms) or calcite (Cal) almost disappeared. Lorentz fitting of FWHM of the kaolinite peak [001] has shown a changing value from 0.17 for the untreated samples to more than 0.50 for the DG-25R ground clay, (Figure 3). Decreased intensities and increased FWHM values suggested a structural disorder and initial amorphization of these phases. FWHM indexes of kaolinite peaks [001] higher than 0.4 suggested a disordered structure of the kaolinite phase [29,30]. The intensity of peaks related to quartz (Qz) did not show significant changes due to MCA process. It can be related to its greater hardness in comparison with kaolinite (7 versus 2–3 in the Mohs scale) which would require application of a significantly longer and more intensive grinding process to induce a visible structural disorder [31]. The number of grinding balls was kept constant, thus changing the B/P ratio altered only the amount of the processed powder. Less powder enabled a more rapid comminution process and, consequently, less time was needed to initiate the MCA. Furthermore, in all cases, the so-called caking, occurred on the inner wall of the jar and on the ball’s surface, leading to problems with the recovery of the processed materials. The effect was more pronounced for samples containing less powder, e.g., DG-25R. Using alcohol or performing brief peptisation helped to avoid these undesirable effects.

**Figure 4:**
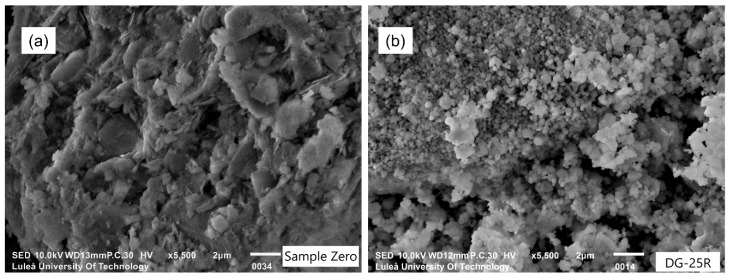
SEM-SE micrographs of a Swedish raw clay: (**a**) sample S0; (**b**) sample DG-25R.

SEM-SE micrographs of the untreated reference sample (S0) and the most reactive sample (DG-25R) are shown in Figure 4. The morphology of the untreated sample consisted of a mixture of platy clay particles and quartz particles. On the contrary, the morphology of the activated sample (DG-25R) was uniform and dominated by spherulitic and quasi-regular particles. Several studies have reported that MCA of kaolin has generated structural changes, as e.g., splitting of the chemical bonds, alteration of the surface and porosity, reduction of the crystal size, etc. [32,33]. Splitting of the O–H, Al–OH, Al–O–Si, and Si–O bonds, and a gradual amorphization of the kaolinite were observed earlier during grinding in a planetary ball mill [34,35].

In the second set of tests, the effects of the B/P ratio during the wet grinding process were investigated by XRD and SEM (Figure 3b). For the dry grinding process, the B/P ratios were 3:1 (WG-3R), 5:1 (WG-5R), and 25:1 (WG-25R) while the W/P ratio was 1:1. In general, changing of the B/P ratio during the wet grinding process showed similar behavior of the XRD curves as during the dry grinding process. The higher the B/P ratio, the more decreased peaks intensities and increased FWHM values for the kaolinite peak [001] were obtained. 

**Figure 5:**
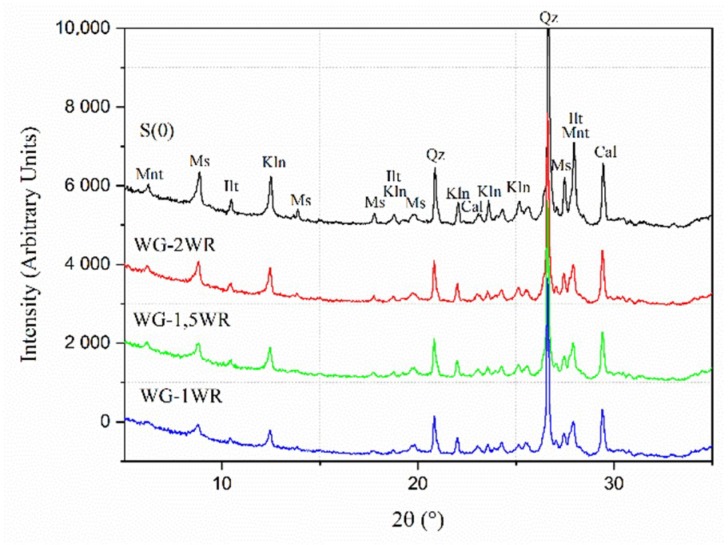
XRD diffraction patterns for different W/P ratio during wet grinding of a Swedish raw clay: S0, WG-1WR, WG-1.5WR, WG-2WR. [Kln-kaolinite, Mnt-Montmorillonite, Ms-muscovite, Ilt-illite, Qz-quartz, Cal-calcite].

In the next step, effects of the W/P ratio were studied. The B/P ratio was fixed at 3:1, which corresponded to a weaker caking effect. The W/P ratios were: 1:1 (WG-1WR), 1.5:1 (WG-1.5WR) and 2:1 (WG-2WR). The grinding time was 15 min. The XRD peaks did not reveal as significant changes as observed in the dry grinding process, Figure 5.

Slightly lower intensities were observed with increasing the W/P ratio. FWHM values did not exceed 0.30 which was earlier suggested as a border value for amorphization [25,26]. This behaviour indicated that a higher amount of water could also lead to an ultimate amorphization but it would require significantly longer grinding times. The presence of water in the system appeared to dilute the suspension and to lower the viscosity. As a result, the generated impact forces were significantly lower. Earlier results showed also that progressing morphological changes tend to affect the effectiveness of the grinding. The effective W/P ratio can decrease, especially in the early stage, leading to elongated processing time [10].

The dry grinding process imposed a rapid decrease of the kaolinite (Kln) peaks intensities, while peaks related to quartz (Qz) were barely affected, (Figure 2a). The wet grinding, on the other hand, appeared to produce strong changes of XRD peaks related to quartz peaks, (Figure 2b). It complies with earlier studies showing more efficient grinding of quartz in the presence of water [31,36,37]. Present results showed traces of chromium (Cr) in samples ground in the presence of water. This contamination can be related to wearing and corrosion of grinding balls and of the inner surface of the jar. In the dry process, the powder surrounding balls and filling the jar tended to act as a protection layer preventing direct impacts. On the contrary, in the wet grinding process, water supported the movement of the powder and prevented the occurrence of the so-called caking effect. It also favored direct impacts between balls and the jar thus increasing the wearing rate. In general, additives, including also water, enable to change the viscosity of the ground material and thus its movement. According to the Rehbinder´s effect, water acting as a foreign adsorbed molecule can increase the viscosity and the specific surface area of the processed solid. Consequently, the resistance to mechanical forces will be decreased [4,38,39]. Furthermore, according to A.Westwood [40], water can also influence the movement of dislocations, their accumulation nearby the surface, and thus it can modify the hardness of the material [4,10].

### 3.2. Grinding Duration

**Figure 6:**
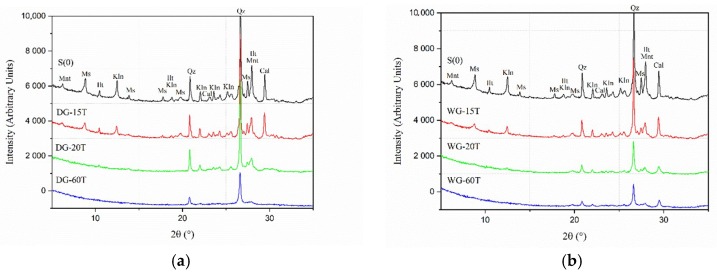
XRD diffraction patterns for different time grinding of a Swedish raw clay (**a**) dry grinding of samples S0, DG-15T and DG-20T, and (**b**) wet grinding of samples S0, WG-15T, WG-20T, and WG-60T. [Kln-kaolinite, Mnt-Montmorillonite, Ms-muscovite, Ilt-illite, Qz-quartz, Cal-calcite].

**Figure 7:**
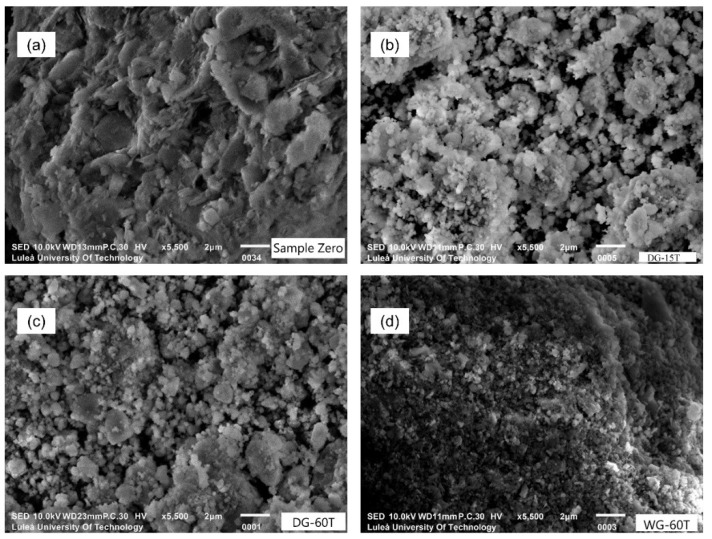
SEM-SE micrographs of processed materials (**a**) sample S0; (**b**) DG-15T; (**c**) DG-60T; (**d**) WG-60T.

The effects of the grinding duration were investigated for both dry and wet processes. Previous tests have shown that the onset of amorphization is accelerated by a higher B/P ratio; therefore, the B/P ratio for these tests was set to 25. In the dry process, the grinding duration was set to: 15 min (DG-15T), 20 min (DG-20T) and 60 min (DG-60T). In the wet grinding, the W/P ratio was 1:1 and the grinding duration was respectively: 15 min (WG-15T), 20 min (WG-20T) and 60 min (WG-60T). In general, based on the intensities of the XRD spectra and on the FWHM values of the kaolinite [001], it can indicate that the amorphization degree was enhanced with longer grinding duration (Figure 6). FWHM indexes for kaolinite [001] were higher than 0,40 for both wet and dry ground samples after 20 min. Additionally, a contamination of the processed material originated from the mechanical wearing of the jar and grinding balls appeared to be present during wet grinding. The wet processed powders had a darker color in comparison with the reference sample (S0) and samples activated with the dry process. SEM-SE observations showed a changing morphology of the processed raw clay (Figure 7).

**Figure 8:**
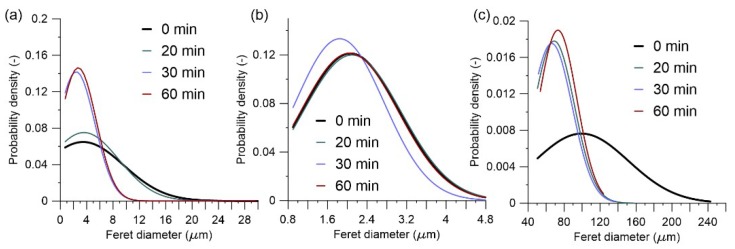
Particle size distribution of a Swedish raw clay at different grinding duration for diameter ranges: (**a**) entire range; (**b**) clay particle size range; (**c**) agglomerates over 50 μm range.

Observations on particle size distribution showed changes in their trend suggesting a correlation with the grinding duration. Prolonged grinding of raw clay produced smaller particles, (Figure 8a). In the clay particles size range (0.98–3.9 μm), the time of grinding did not show significant changes (Figure 8b), even if the chemical reactivity of the material was evidently changed according to the alkali activation of the same samples. Grinding modified the agglomerates size distribution, as shown in the Figure 8b. Earlier studies also confirmed that particle size distribution changed mostly in the first hour of grinding and depends on the grinding equipment parameters [35]. 

### 3.3. Grinding Speed

**Figure 9:**
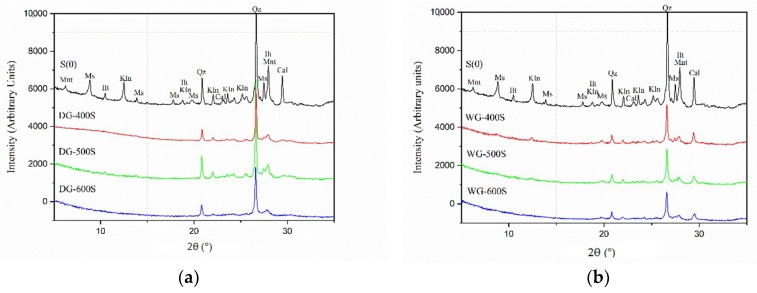
XRD diffraction patterns for different speeds during grinding of a Swedish raw clay: (**a**) dry grinding for sample S0, DG-400S, DG-500S and DG-600S; and (**b**) wet grinding for sample S0, WG-400S, 500 rpm WG-500S, and WG-600S. [Kln-kaolinite, Mnt-Montmorillonite, Ms-muscovite, Ilt-illite, Qz-quartz, Cal-calcite].

**Figure 10:**
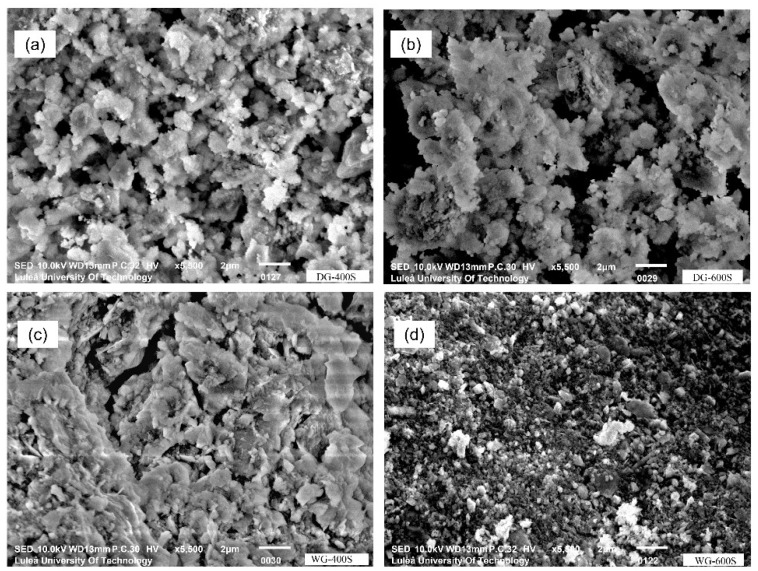
SEM-SE micrographs of Swedish raw clay for samples: (**a**) DG-400S; (**b**) DG-600S; (**c**) WG-400S; (**d**) WG-600S.

**Figure 11:**
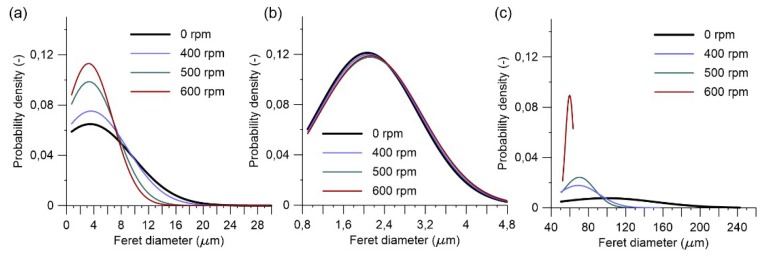
Particle size distribution of a Swedish raw clay at different grinding speeds for diameter ranges: (**a**) entire range; (**b**) clay particle size range; (**c**) agglomerates over 50 μm range.

Effects of the grinding speed were evaluated for both dry and wet processes, including 400, 500 and 600 rpm, Figure 9 and Figure 10. In all the cases, a higher rotation speed increased the temperature of the grinded material. In addition, higher speeds applied in the wet grinding produced finer particles and created pastes having a higher viscosity. As a result, grinding balls were fully immobilized and the activation process was completely hindered after a short time. The removal of the grinded material was extremely difficult. The amorphization evaluated by the decreased peaks and FWHM indexes from the XRD diagrams (Figure 10), showed a generally enhanced amorphization with the increased grinding speed for both processes. However, the maximum used speed of 600 rpm, which induced extensive amorphization, also caused a strong caking effect. The amorphization of kaolinite, muscovite and calcite was significantly increased at higher speeds in the case of both wet and dry processes. SEM analysis detected morphological changes, Figure 11.

Particle size distribution was investigated also for different grinding speeds. Fewer agglomerates and a particle size distribution with increased amount of finer particles were observed with increasing grinding speeds (Figure 11a). Ground clay with higher rotation speed produced smaller agglomerates (Figure 11c). The dis-agglomeration seemed to be affected by the rotation speed parameter of the grinding equipment. Regarding the smaller particles present in the clay, there was no evident change in their size (Figure 11b).

### 3.4. Alkali Activation of Processed Material—Preliminary Tests

**Figure 12:**
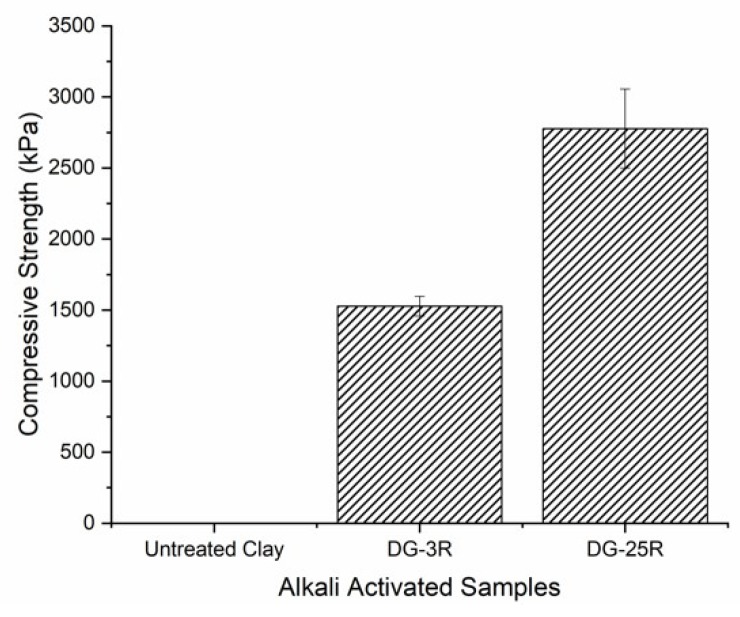
7-days compressive strength results of alkali-activated samples: S0, DG-3R and DG-25R.

Mortar samples produced with untreated clay did not show any mechanical strength indicating a lack of chemical activity. On the contrary, samples produced with the MCA activated clay reached 1500 kPa and 2700 kPa after 7 days of sealed curing, respectively for the DG-3R and DG-25R and Figure 12. These results clearly indicate induced chemical reactivity during the MCA process, which increased with higher degree of amorphization as determined by the XRD analysis. Future research will be continued on optimization of the MCA process and on the alkali activation.

## 4. Conclusions

The effects of grinding media and grinding duration on the amorphization and morphological changes of the raw Swedish clay were studied. The following main conclusions were formulated:

- The effectiveness of the mechanochemical activation in the early stage of grinding (within the Rittinger’s zone), is very sensitive to changes of the ball to powder ratio, the amount of the grinding medium, the water to powder ratio and the speed regime of the equipment.
- Dry grinding process promoted more extensive amorphization when using a higher number of grinding balls versus the amount of the processed clay powder.
- Dry grinding was more effective in amorphization of kaolinite than wet grinding process.
- Effectiveness of the wet grinding increased with a higher water to processed powder ratio but required longer processing times in comparison with the dry grinding process.
- Longer grinding enabled a more extensive amorphization in both wet and dry processes, produced finer particles with spherulitic morphology, but also tended to increase agglomeration when outside of the Rittinger zone.
- Higher rotational speed enhanced amorphization efficiency of clay minerals in both wet and dry processes. The trend was similar in the case of quartz but only when the wet grinding was used.
- The most efficient amorphization of the studied Swedish raw clay was achieved when using the following process parameters: B/P ratio equal to 25, speed 500 rpm and the grinding time of 20 min. This process also caused less agglomeration, caking effect and wearing.
- The processed raw clay, using the defined parameters, should enable its usage as pozzolanic material and as cementitious binder for production of concrete.
- Preliminary strength tests validated the concept that the MCA can enhance the reactivity of the raw clay to a degree enabling its utilization as a binder in alkali-activated systems for e.g., production of mortars or concretes.

## Figures and Tables

**Table 1:** Chemical composition of the raw clay.

Component	Content (wt.%)
SiO_2_	52.6
Al_2_O_3_	15.1
Fe_2_O_3_	6.9
CaO	6.41
K_2_O	3.78
MgO	2.51
Na_2_O	1.68
TiO_2_	0.696
LOI	7.5

**Table 2:** Studied process parameters.

Water/Powder	Balls/Powder	Speed (rpm)	Grinding Duration (min)	Sample ID.
-	3	500	20	DG-3R
	5			DG-5R
	25			DG-25R
-	25	500	15	DG-15T
			20	DG-20T
			60	DG-60T
-	25	400	20	DG-400S
		500		DG-500S
		600		DG-600S
1	3	500	20	WG-3R
	5			WG-5R
	25			WG-25R
1	3	500	15	WG-1WR
1.5				WG-1,5WR
2				WG-2WR
1	25	500	20	WG-20T
			30	WG-30T
			60	WG-60T
1	25	400	20	WG-400S
		500		WG-500S
		600		WG-600S

**Table 3:** Mix composition of Alkali-Activated raw clay.

	Mix Identification		
	Untreated Clay (S0)	DG-3R	DG-25R
Binder content wt. (g)	30	30	30
Water/Binder (ratio)	0.5	0.5	0.5
Aggregate content wt. (g)	30	30	30
Wt.% alkali activator solution	10	10	10
Activator Modulus Ms (SiO_2_/Na_2_O)	1	1	1
